# Lipid storage myopathy due to late-onset multiple Acyl-CoA dehydrogenase deficiency with novel mutations in ETFDH: A case report

**DOI:** 10.3389/fneur.2022.991060

**Published:** 2022-12-15

**Authors:** Huihong Tian, Yi Zhong, Zhihua Liu, Liping Wei, Yanbo Yuan, Yuhu Zhang, Limin Wang

**Affiliations:** ^1^Department of Neurology, Guangdong Provincial People's Hospital, Guangdong Neuroscience Institute, Guangdong Academy of Medical Sciences, Guangzhou, China; ^2^Shantou University Medical College, Shantou, China; ^3^South China University of Technology School of Medicine, Guangzhou, China; ^4^The Second School of Clinical Medicine, Southern Medical University, Guangzhou, China

**Keywords:** lipid storage myopathy, multiple acyl-CoA dehydrogenase deficiency, ETFDH, riboflavin, whole genome sequencing

## Abstract

**Background:**

Lipid storage myopathy (LSM) is an autosomal recessive inherited lipid and amino metabolic disorder with great clinical heterogeneity. Variations in the electron transfer flavoprotein dehydrogenase (ETFDH) gene cause multiple acyl-CoA dehydrogenase deficiency (MADD), and have a manifestation of LSM. Muscle biopsy helps clarify the diagnosis of LSM, and next-generation sequencing (NGS) can be useful in identifying genomic mutation sites. The diagnosis of MADD contributes to targeted therapy.

**Case presentation:**

We report on a teenager who appeared to have muscle weakness and exercise intolerance at the onset. Before the referral to our hospital, he was unsuccessfully treated with glucocorticoid for suspected polymyositis. The next-generation sequencing of the proband and his parents revealed heterozygous variations, c.365G>A (p.G122D) inherited from the father, c.176-194_176-193del, and c.832-316C>T inherited from the mother in the ETFDH gene. The tandem mass spectrometry identified the mutations to be pathogenic. However, his parents and his younger sister who were detected with a mutation of c.365G>A presented no clinical symptoms. This indicates that the combination of the three compound heterozygous mutations in ETFDH is significant. After MADD was diagnosed, a dramatic clinical recovery and biochemical improvement presented as riboflavin was given to the patient across a week, which further confirmed the diagnosis of MADD.

**Conclusion:**

Our observations extend the spectrum of ETFDH variants in Chinese the population and reinforce the role of NGS in diagnosis of MADD. Early diagnosis and appropriate treatment of LSM lead to great clinical efficacy and avoid some lethal complications.

## Introduction

Lipid storage myopathy (LSM) is an inherited autosomal recessive disease caused by disorders in lipid and amino acid metabolism (1). Multiple acyl-CoA dehydrogenase deficiency (MADD), also called glutaric aciduria type II, is the most common type and accounts for ~90% of LSM in China. Other types of LSM include primary carnitine deficiency (PCD), neutral lipid storage disease with ichthyosis (NLSDI), and neutral lipid storage disease with myopathy (NLSDM) (2). The pathologic manifestation is deposition of lipid droplets in muscle fibers (3). MADD is related to mutations in genes encoding the electron transfer flavoprotein alpha and beta (ETFA and ETFB) proteins or the electron transfer flavoprotein dehydrogenase (ETFDH) protein (4). MADD could be simply divided into neonatal-onset and late-onset MADD. Ninety percent of late-onset MADD is caused by mutations in ETFDH, while neonatal-onset MADD is related to ETFA or ETFB (5).

Herein, we discuss a young male patient who complained of muscle weakness and exercise intolerance. Three novel compound heterozygous mutations are located in one exon and two introns. He was misdiagnosed with polymyositis and treated with a glucocorticoid, resulting in worse parameters of muscle enzyme. After confirmation of the diagnosis of LSM (MADD), the patient received riboflavin replacement therapy accompanied by coenzyme Q10 and levocarnitine, and had a full recovery.

## Case report

A 17-year-old boy was admitted to our hospital because of progressive muscle weakness and exercise intolerance for over 6 months. Initially, he had difficulty standing up from squatting and climbing stairs. He had to stop to rest when climbing three to four floors. On admission, he could only climb one floor or walk 20–30 m. Gradually, his upper extremities and jaw were also affected; he could not lift heavy weights and had trouble chewing. He was diagnosed with inflammatory myopathy and treated with corticosteroid in the first hospital. However, his symptoms were not alleviated. For all we know, his siblings and parents did not present similar symptoms.

The physical examination showed moderate muscle weakness (grade 3/5 for the proximal lower limbs, grade 4/5 for the distal lower limbs, grade 4/5 for the upper limbs) according to the Medical Research Council scale, he had a waddling gait, and there was no knee jerk reflex. Cranial nerves were examined and found to be normal including functions of chewing and swallowing. On admission, myocardial enzyme levels were elevated markedly, and creatine kinase (CK), lactate dehydrogenase (LDH), hydroxybutyrate-dehydrogenase (HBDH) were 2,387 (range 50–310 U/L), 1,185 (range 120–250 U/L), and 1,049 U/L (range 72–182 U/L), respectively. Alanine aminotransferase (ALT) was 85 U/L (range 9–50 U/L), and aspartate aminotransferase (AST) was 178 U/L (range 15–40 U/L). Uric acid was 640 umol/L (range 208–428). Rheumatic immune indexes were negative. Although one of the eight myositis antibodies related to idiopathic inflammatory myopathies, antialanyl tRNA synthetase (PL-12), was positive, we think it was not clinically significant for the following reasons. The patient did not have muscle pain and tenderness, and the muscle biopsy did not show inflammatory infiltrates; what is more, he had no response to the glucocorticoid treatment. Antialanyl tRNA synthetase (PL-12) was positive, while other immunity indexes were negative. The electromyography revealed myogenic damage, while the magnetic resonance imaging showed no abnormalities of the bilateral quadriceps femoris. The magnetic resonance imaging of the head was normal, and the electroencephalogram was approximately normal. An abdominal ultrasound scan demonstrated that he had a fatty liver.

**Figure 1:**
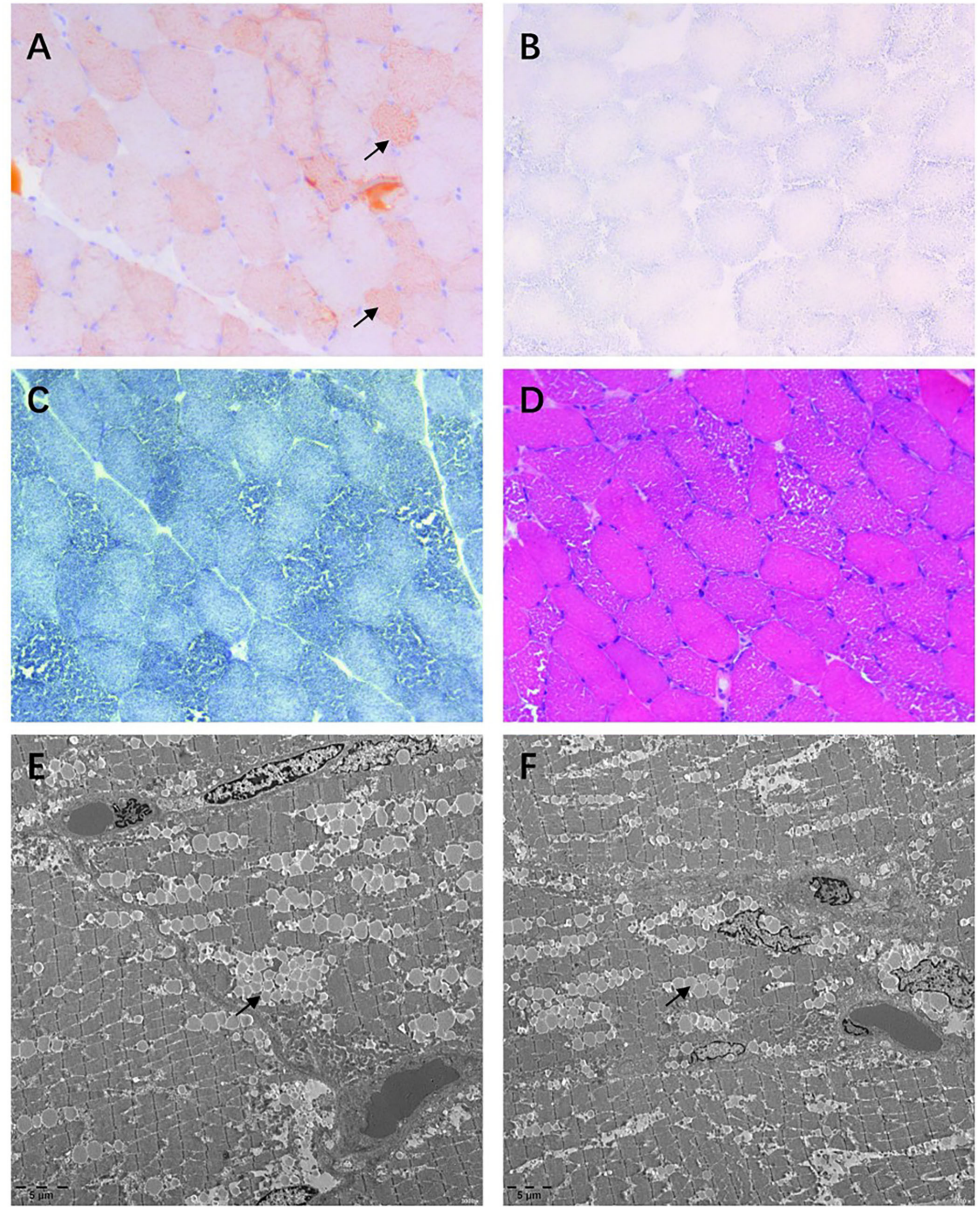
Muscle biopsy of the quadriceps femoris. **(A)** Oil Red O (ORO) stain showing lipid droplets deposited in some muscle fibers. **(B,C)** Lipid droplet is almost invisible in succinate dehydrogenase (SDH) and nicotinamide adenine dinucleotide (NADH) stains. **(D)** Hematoxylin and eosin (H and E) stain demonstrating abundant vacuoles appearing in most fibers. **(E,F)** Considerable lipid droplets are observed by transmission electron microscopy.

Muscle biopsies of the biceps brachii and quadriceps femoris revealed increased deposition of lipid droplets in muscle fibers stained with Oil Red O (ORO) (Figure 1A), which indicated lipid storage myopathy (Figure 1). However, succinate dehydrogenase (SDH) (Figure 1B) and nicotinamide adenine dinucleotide-tetrazolium reductase (NADH-TR) (Figure 1C) were negative for fat vacuole deposition. The hematoxylin and eosin (H and E) (Figure 1D) stain showed a little inflammatory cell infiltration, and abundant small vacuoles were observed in a large number of muscle fibers. Electron microscopy demonstrated that lipid droplets increased in subsarcomembrane and of myofibrils.

The genetic testing (next-generation sequencing) of the boy and his parents confirmed novel compound heterozygous mutations of the ETFDH gene: (OMIM:231675, NM_004453), c.365G>A (p.G122D), c.176-194_176-193del, and c.832-316C>T, which were predicted to be deleterious in chromosome 4. The exome sequencing of his father showed a compound heterozygous mutation, c.365G>A (p.G122D), in exon 4, while his mother carried compound heterozygous mutations, c.176-194_176-193del and c.832-316C>T, in intron 4. The result of the gene test showed that the mutations of ETFDH in the proband came from his patients and were consistent with the pattern of autosomal recessive inheritance. His 6-year-old sister was asymptomatic, and her NGS test showed mutation of c.365G>A (p.G122D), which was inherited from the father. None of the mutations were reported in previous studies; that is, the mutations of the proband are not identified in the Human Gene Mutation Database (HGMD). Meanwhile, the significance of these mutations is not clear. To solve this problem, tandem mass spectrometry and gas chromatography-mass spectrometry of urinary organic acids was conducted in the 1-year follow-up, and we found that the mutations had pathogenicity. To the best of our knowledge, this is the first report on three compound heterozygous variants of ETFDH involved in a patient. In addition, this is the first study that reports gene mutation in a intron, which may benefit from the development of technology.

After confirming the diagnosis of MADD, a combination of riboflavin (60 mg/day), levocarnitine (30 ml/day), and CoQ10 (30 mg/day) was administered to the patient with great improvement in muscle weakness and exercise intolerance in 1 week. In addition, his CK and CK-MB levels were obviously decreased (CK: 5,023 to 864 U/L, CK-MB: 129 to 37 U/L) after having taken the medicines for 4 days. However, CK and CK-MB rose to 9,204 and 70.5 U/L, respectively, in 1 month because of the withdrawal of drugs aalone. The patient adhered to the medication for the next 2 months, the muscle weakness and exercise intolerance completely disappeared, and the creatine kinase values went back to normal (CK: 123 U/L, CK-MB: 11 U/L), as well as the other myocardial enzymes (LDH: 184 U/L, HBDH: 131 U/L, ALT: 34 U/L, AST: 22 U/L). Besides, his uric acid level was decreased to 500 umol/L, a near-normal value. Except for the medications, the patient adhered to a low-fat, high-protein, and low-purine diet. In addition, he did regular low-intensity exercises after discharge. The patient was re-examined by electromyography 1 year post incidence, which suggested myogenic damage but the manifestations of muscles were better than before.

## Discussion

Late-onset MADD is mainly caused by homozygous or compound heterozygous mutations in the ETFDH gene. According to previously published articles, several mutation sites are the most common spots: c.250G > A (p.A84T), c.770A > G (p.Y257C), and c.1227A > C (p.L409F) (6, 7).

In this case, the patient was diagnosed with hyperuricemia in the first hospital, but his uric acid decreased gradually after taking riboflavin without using a uric acid-lowering drug. A study involving eight patients with LSM complicated by hyperuricemia found that men with LSM are more likely to develop hyperuricemia than women with LSM because of the benefit of estrogen (8). The cause of hyperuricemia is mainly related to decreased uric acid excretion, which could be explained by renal tubular disorder. The article points out that in young patients who experience muscle weakness and hyperuricemia, LSM should be considered as a differential diagnosis.

Previous literature found that the clinical manifestation of patients with later-onset MADD often involves hepatomegaly, encephalopathy, rhabdomyolysis, vomiting, hypoglycemia, and so on (7, 9–11). Symmetric proximal muscle weakness of the lower extremities was the main complaint of the patient. He was diagnosed with fatty liver by the abdominal ultrasound scan despite being a teenager and keeping a healthy lifestyle. We suspected that his fatty liver was related to MADD.

Before his diagnosis of LSM, low-dose dexamethasone had been used for a week, resulting in worse outcomes of muscle enzyme and no improvement in muscle strength. It may be because the disease continued to progress and the treatment was useless. However, a case reported that a young woman with proximal tetraparesis and myalgia was diagnosed with MADD and had a surprising response to treatment of plasmapheresis and intravenous immunoglobulin (IVIg) (12). It indicates that guidelines for treatment should be refined. Except for invasive muscle biopsy, muscle magnetic resonance imaging can also be conducted to distinguish between MADD and immune-mediated necrotizing myopathy (13). The MRI of the quadriceps femoris muscle of this patient revealed no abnormality, this result may be attributed to the short duration. The mechanism of how immunomodulatory treatment works is not clear. Therefore, further research on the pathophysiology of MADD is needed.

The diagnosis of MADD is based on combination of clinical presentations, acylcarnitines and urine organic acids, muscle biopsy, and genetic analysis. Muscle biopsy is the main criterion for diagnosis of LSM, and genetic diagnosis is conducive to a more accurate diagnosis and future gene-targeted therapy. Meanwhile, muscle MRI can be a complementary and differential examination technique, as it is non-invasive. In this case, because of his symptom of muscle weakness and the positive myositis-specific autoantibody, anti-alanyl tRNA synthetase (anti-PL-12), we gave him dexamethasone as a diagnosis of polymyositis was considered, but it had little clinical efficacy. The biopsy confirmed the diagnosis of LSM, and the gene screening confirmed MADD through mutations of ETFDH. Almost all cases of late-onset MADD is related to variants of the ETFDH gene and riboflavin response (6). MADD is potentially lethal but curable after timely treatment with full-dose riboflavin. Thus, it is essential to diagnose the disease as early as possible. The limitation of our study is that it is hard to define which drug mainly produced a curative effect while three drugs were being applied simultaneously, namely, riboflavin, coenzyme Q10, and levocarnitine. According to previous studies, a dosage of 100–400 mg/d of riboflavin is needed (1). However, the patient was relieved with only 60 mg of riboflavin and CoQ10, and L-carnitine. The use of CoQ10 and L-carnitine in MADD is controversial. MADD may cause secondary carnitine and CoQ 10 deficiency (14).

## Conclusion

We have identified a novel compound heterozygous mutation in the ETFDH gene from a patient with late-onset riboflavin-responsive multiple acyl-CoA dehydrogenase deficiency, which expands the spectrum of mutations found in patients with MADD. Late-onset MADD is a curable hereditary disease. Besides, early diagnosis and treatment could reduce complications, so this article can help improve the knowledge of LSM.

## Data availability statement

The datasets presented in this article are not readily available because of ethical and privacy restrictions. Requests to access the datasets should be directed to the corresponding author.

## Author contributions

All authors listed have made a substantial, direct, and intellectual contribution to the work and approved it for publication.
